# HER2-low breast cancer: evolution of HER2 expression from primary tumor to distant metastases

**DOI:** 10.1186/s12885-023-11134-4

**Published:** 2023-07-13

**Authors:** Mengyuan Cai, Ming Li, Hong Lv, Shuling Zhou, Xiaoli Xu, Ruohong Shui, Wentao Yang

**Affiliations:** 1 Department of Pathology, Fudan University Shanghai Cancer Center, Shanghai, 200032 China; 2 Department of Oncology, Shanghai Medical College, Fudan University, Shanghai, 200032 China; 3 Institute of Pathology, Fudan University, Shanghai, 200032 China

**Keywords:** HER2-low expression, HER2 discordance, Metastasis, Anti-HER2 antibody-drug conjugates, Breast cancer

## Abstract

**Background:**

Breast cancer (BC) with low human epidermal growth factor receptor 2 (HER2) expression is attracting much attention due to the breakthrough progress of novel anti-HER2 antibody-drug conjugates. HER2 expression is examined in patients with HER2-low BC and their distant metastases in this study, so as to further clarify the dynamic characteristics of HER2 low status in the process of disease progression.

**Methods:**

Patients diagnosed with HER2 low breast cancer (defined as IHC1+ or IHC2+/ISH-) between 2012 and 2021 were included in this study. We evaluated HER2 expression of primary sites and metastatic sites, compared the impact of different clinicopathological parameters on HER2 status of metastases and compared the overall survival and disease-free survival of patients with different HER2 status in metastases.

**Results:**

Ninety-eight patients were included. All HER2 IHC scores were confirmed and the consistent rate with the original pathological report was 81.1%. 27.6% of the patients showed different HER2 status in metastases. The HER2 discordance rate differed among different metastatic sites (*p* = 0.040). The higher the T stage of the primary BC, the higher the rate of HER2 discordance was observed (*p* = 0.042). For the specimen type of metastasis, HER2 discordant rate was higher in surgical specimen than biopsy (*p* = 0.050). No difference of HER2 discordance rate was found between HER2-1+ and HER2-2+ patients. But comparing HER2 IHC score, HER2-2+ patients were less likely to have consistent metastatic HER2 levels than HER2-1+ patients (*p* = 0.006). No difference in survival outcomes was observed between patients with different HER2 status in metastases.

**Conclusions:**

There is a possibility of HER2 expression alteration in the metastases of HER2-low breast cancer. And the rate of altered HER2 low expression was different among different metastatic sites, different T stages of primary BC and specimen type of metastasis. No prognostic significance was observed.

**Supplementary Information:**

The online version contains supplementary material available at 10.1186/s12885-023-11134-4.

## Background

As one of the most frequently diagnosed cancer in the world [1], Breast cancer (BC) is a highly heterogenous disease, with significant biological diversity and different clinicopathologic features, prognosis and sensitivity to treatments. Despite complex biological diversity, in clinical practice breast cancer can be divided into several subgroups according to estrogen receptor (ER), progesterone receptor (PR) and human epidermal growth factor receptor 2 (HER2) status. For anti-HER2 targeted agents, the treatment-decision is usually made according to dichotomization. In the context of HER2-positive BC (which is defined as HER2 3+ on IHC(immunohistochemistry) score or HER2 gene amplification on ISH(in situ hybridization) assay [2]), which accounts for about 15% of all BCs [3], anti-HER2 agents has shown great clinical benefits [4, 5]. While HER2-negative BCs, the definition of which is IHC 0/1+ or IHC 2+ with no HER2 gene amplification [2], have been proved with no benefit from traditional HER2-targeted agents by several studies [6–8].

Recently, however, results from several clinical trials focusing on new anti-HER2 antibody-drug conjugates (ADCs) in breast cancer patients with HER2-low expression (defined as IHC1+ or IHC2+ without HER2 gene amplification by ISH) are changing the situation [9–11]. For instance, the results of a phase Ib study showed that Trastuzumab Deruxtecan (DS8201a) achieved a remarkable 10.4 months of median response duration and 37% of overall response rate in patients with HER2-low advanced breast cancer [9]. Likewise, in HER2-low breast cancer patients with hormone receptor positive (HR+) or HR-, SYD985 (Trastuzumab Duocarmazine) had an objective response rate (ORR) of 28% and 40% separately in phase Ib trials [10]. And the results of Destiny-Breast04, a phase III trial testing DS8201a in pretreated advanced HER2-low breast cancer patients showed that the median progression free survival (PFS) was 4.8 months longer in the DS8201a group compared with 5.1 months in the physician’s choice group (*p* < 0.001), and the overall survival (OS) was 6.6 months longer (*p* = 0.001) [11].

Based on all these results, HER2-low breast cancer is becoming a new important entity different from the old HER2-negative group. And several reports have investigated that the proportion of HER2-low breast cancer in the population is about 45–55% [12–14], which is not negligible. It has been reported that there exists HER2 status difference between primary and recurrent breast cancer [15–17]. And for HER2-low breast cancer, this phenomenon also has been reported [18, 19]. Nonetheless, most of the reported studies are about patients with HER2-neagtive breast cancer and the changes of HER2 expression in recurrent or advanced stage [18, 19], and few evidence has yet been reported concerning the evolution of HER2 status focusing on the HER2-low primary breast cancer and the matched distant metastasis.

In this study, our goal is to characterize the HER2 status of distant metastasis in patients with HER2-low primary breast cancer, evaluate the association of the HER2 status discordance with the clinicopathologic features and tumor-related factors, as well as exploring the possible prognostic value.

## Methods

### Sample selection

Breast cancer patients who had received biopsy or surgical resection of both primary lesion and distant metastasis at Fudan University Shanghai Cancer Center from July 4, 2012 to October 13, 2021 with available HER2 IHC score and, if necessary, HER2 FISH (fluorescence in situ hybridization) results of both primary and metastatic tumor were retrieved from a pathology database. HER2 IHC slides of all primary and metastatic tumor samples were retrieved from the Pathology Departments of our center. Patients clinicopathologic features including HER2 status, HR status, age, histological subtype and grade, site and timing of distant metastases, and survival status were recorded.

### Evaluation of HER2 expression

All staining was performed by the auto-staining machine, which is Ventana BenchMark autostainer or Ventana BenchMark Ultra autostainer (Ventana Medical System Inc, Roche, Tucson, Arizona) with the BenchMark ULTRA advanced staining system operator guide. ULTRA Cell Conditioning Solution (ULTRA CC1, pH = 8.5) was used to perform antigen retrieval at 90 °C to 100 °C. HER2 expression was all tested by prediluted Ventana 4B5 antibody, and was reassessed by a pathologist according to the latest standard of HER2 diagnosis by ASCO/CAP Guidelines Update [2], and cases that were difficult to diagnose were reassessed by another pathologist. Tumors were considered HER2-low when the IHC score was 1+ or 2+ with negative ISH assay. And according to the latest ASCO/CAP Guidelines Update [2], IHC 0 is defined by no staining observed or membrane staining that is incomplete and is faint or barely perceptible and within ≤ 10% of the invasive tumor cells, and if the proportion is over 10%, tumor is defined as IHC 1+. The definition of IHC 2+ is invasive breast cancer with weak to moderate complete membrane staining observed in > 10% of tumor cells, and when over 10% of circumferential membrane staining is complete and intense, tumor is defined as IHC 3+.

### Statistical analysis

SPSS 20.0 statistical software (IBM Corp, Armonk, NY, USA) was used to perform statistical analysis., For continuous variables, data was presented as median and interquartile range, and for categorical variables, data was presented as relative frequencies (percentage). Chi-square and Fisher’s exact tests were used to investigate differences by HER2-low status for categorical variables and Wilcoxon rank test for continuous variables. Kaplan-Meier curves were performed using GraphPad Prism 9.0 for survival analysis. *P* < 0.05 was considered statistically significant.

## Results

### Patients and their clinicopathologic features

**Fig. 1:**
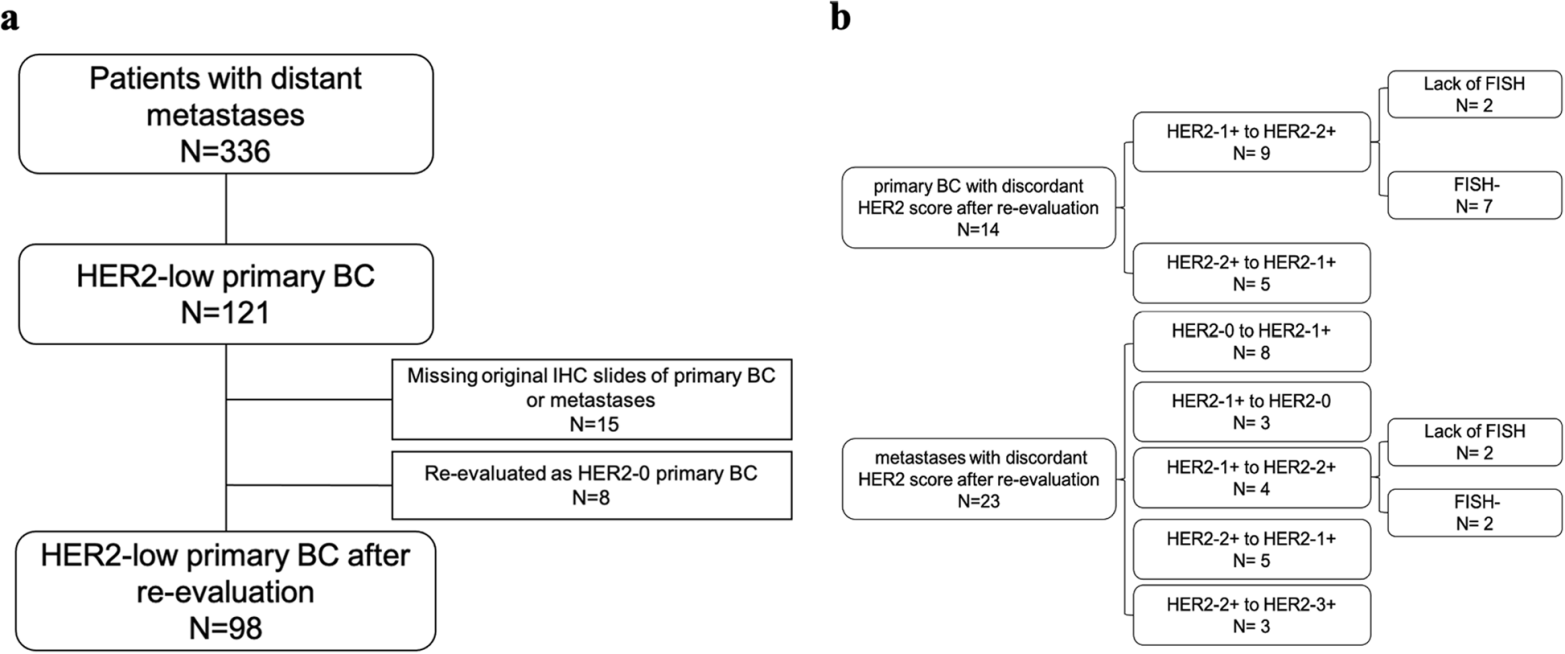
Case selection and grouping. **a** Flow diagram of the study. **b** Re-evaluation results of the HER2 IHC score. N number, BC breast cancer, IHC immunohistochemistry, FISH fluorescence in situ hybridization

**Fig. 2:**
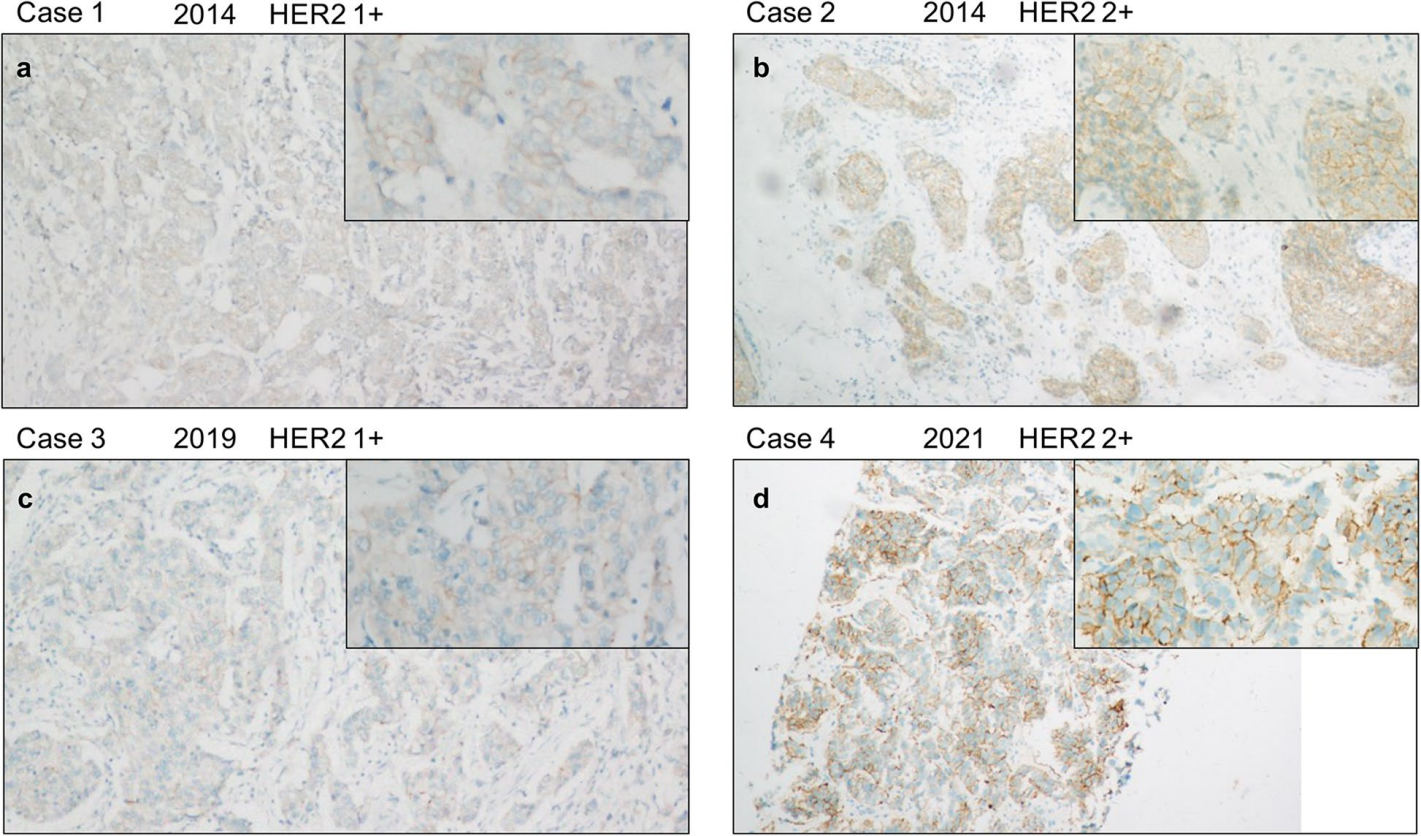
HER2 staining situation of different years. Case 1 (**a**) showing HER2 1+ staining situation of 2014. Case 2 (**b**) showing HER2 2+ staining situation of 2014. Case 3 (**c**) showing HER2 1+ staining situation of 2019. Case 4 (**d**) showing HER2 2+ staining situation of 2021. All of the insets are high-power field highlighting area of HER2 immunoreactivity

Three hundred thirty-six patients with available HER2 IHC score and, if necessary, FISH results on both primary BC and distant metastases were included during 2012 and 2021. Among these cases, 121 patients with HER2-low primary BC were selected. 15 cases lacking original HER2 IHC slides were ruled out. And 8 cases re-evaluated as HER2-0 of primary BC were ruled out (Fig. 1a). In total, 98 patients with HER2-low BC who had distant metastases were included in the analysis, including 55 (56.1%) patients with HER2-1+ and 43 (43.9%) patients with HER2-2+. And among the overall population, 78.6% of the patients were HR+, and 21.4% were triple negative breast cancer (TNBC). Table 1 summarized the clinical, pathological and immunohistochemical characteristics. All of the HER2 IHC-stained slides were rescored, and the concordance rate of HER2 IHC score with the original pathological report was 81.1% (Fig. 1b). For primary BC with HER2-low expression, the concordance rate between HER-1+ and HER2-2+ was 85.7%. And for metastatic sites, the concordant rate among different HER2 IHC scores was 76.5%. And 4 cases with HER2-1+ were re-evaluated as HER2-2+ which lacked FISH results. Considering their relatively weak HER2 staining, they were still classified as HER2-low for subsequent statistical analysis. For the possible fading of DAB staining, we also compared IHC slides of different years, and we believed that the fading didn’t affect the HER2 diagnosis (Fig. 2).

**Table 1:** Clinical, pathological and immunohistochemical characteristics of HER2 low breast cancers

Variable	Overall (98)	HER2-1+ (55)	HER2-2+ (43)	*P*-value
	N(%) or mean	N(%) or mean	N(%) or mean	
Age at primary diagnosis/y	50.5 ± 11.6	50.3 ± 11.3	51.7 ± 11.6	0.552
Tumor size				0.089
- ≤ 2 cm	22 (22.4)	16 (29.1)	6 (14.0)	
- > 2 cm	47 (48.0)	24 (43.6)	23 (53.4)	
-Unavailable	29 (29.6)	15 (27.3)	14 (32.6)	
Histological subtype				1.000
-Invasive breast carcinoma NOS	93 (94.9)	52 (94.5)	41 (95.3)	
-Others	5 (5.1)	3 (5.5)	2 (4.7)	
Histological grade				0.459
-II	52 (51.0)	31 (56.4)	21 (48.8)	
-III	46 (45.9)	24 (43.6)	22 (51.2)	
Estrogen receptor expression				0.697
-Positive	77 (78.6)	44 (80.0)	33 (76.7)	
-Negative	21 (21.4)	11 (20.0)	10 (23.3)	
Progesterone receptor expression				0.579
-Positive	60 (61.2)	35 (63.6)	25 (58.1)	
-Negative	38 (38.8)	20 (36.4)	18 (41.9)	
Ki67				0.371
- < 20%	15 (15.3)	10 (18.2)	5 (11.6)	
- ≥ 20%	83 (84.7)	45 (81.8)	38 (88.4)	
Age at metastasis diagnosis	53.0 ± 11.7	52.2 ± 11.3	53.9 ± 11.8	0.471
Site of metastasis				0.929
-Bone	13 (13.3)	8 (14.5)	5 (11.6)	
-Lung/Pleura	28 (28.6)	15 (27.3)	13 (30.2)	
-Liver	41 (41.8)	24 (43.6)	17 (39.5)	
-Skin and soft tissues	11 (11.2)	6 (10.9)	5 (11.6)	
-Others	5 (5.1)	2 (3.6)	3 (7.0)

### Discordance between primary breast cancer and distant metastases regarding HER2 status

**Fig. 3:**
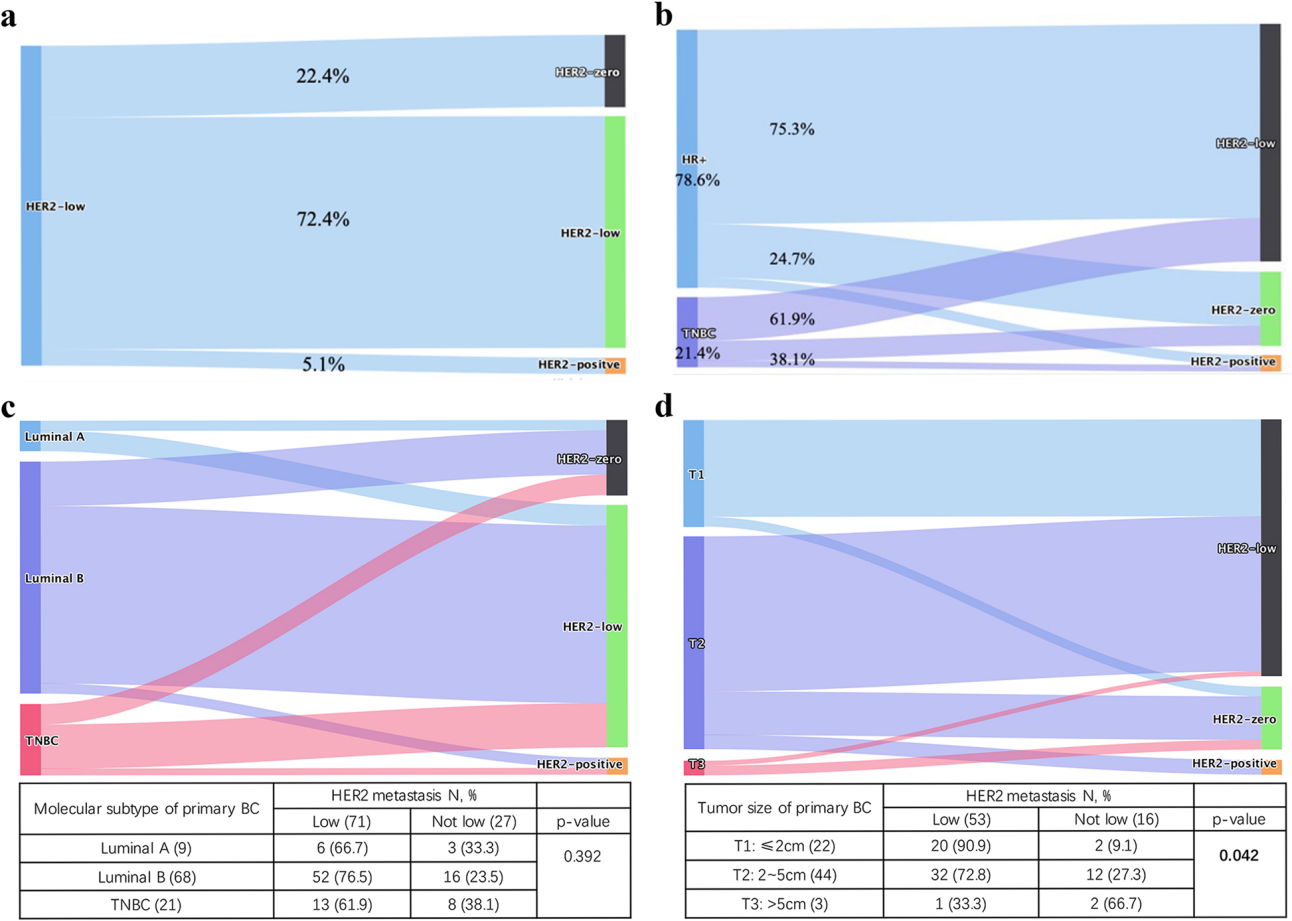
HER2 evolution from primary breast cancer to distant metastases. **a** The overall rate of HER2 discordance. **b** HER2 discordant cases in HR-positive and triple-negative breast cancers respectively. **c** Evolution of the HER2 status between primary tumors and metastases stratified by molecular subtypes of primary BC (Chi-square test). **d** Evolution of the HER2 status between primary tumors and metastases stratified by T stages of primary BC (Fisher’s exact test). BC breast cancer, N number

**Fig. 4:**
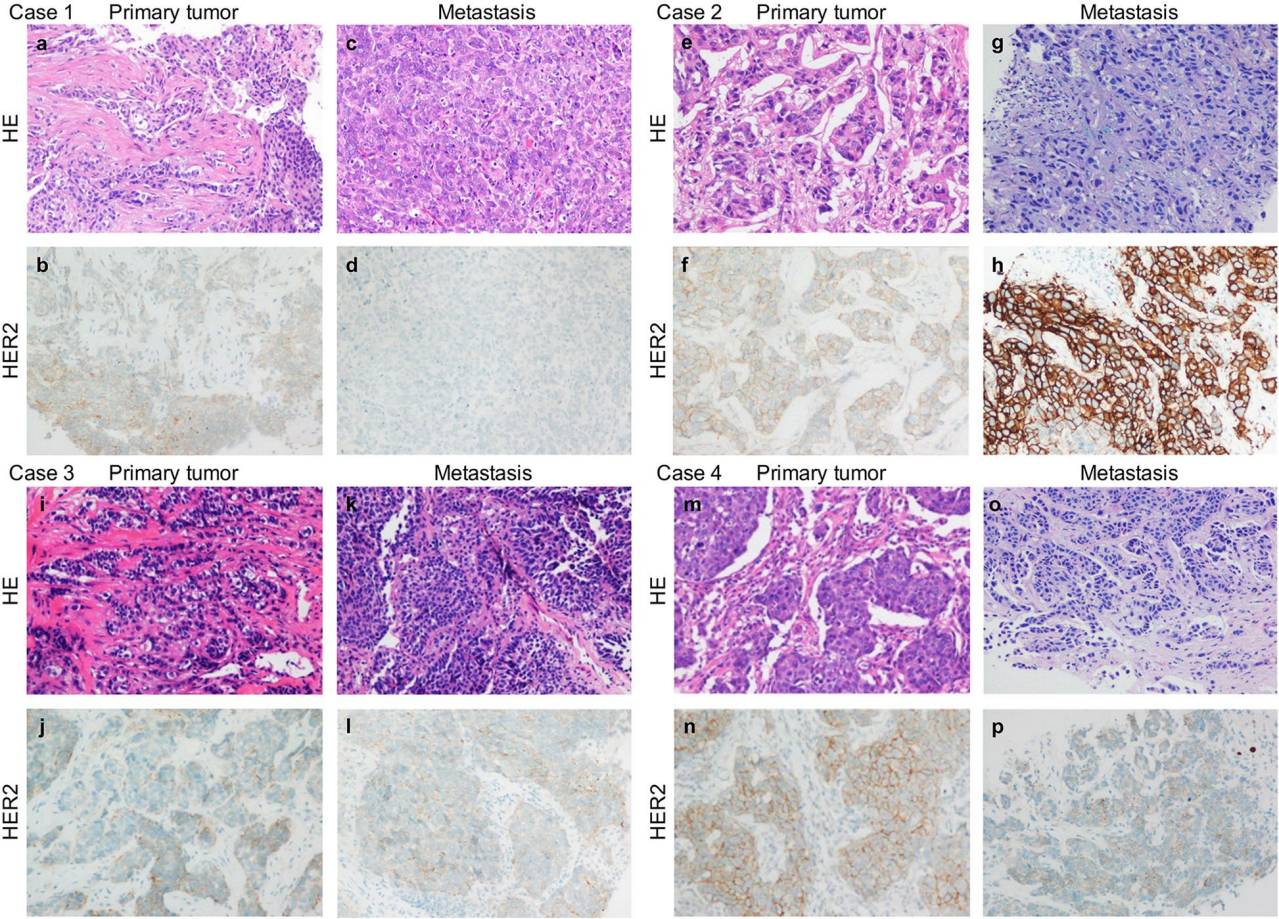
Four examples of HER2 status evolution from primary breast cancer to distant metastases. High-power views of four primary breast carcinomas and their metastases, with corresponding HER2 IHC images. Case 1: a HER2-low (1+) primary tumor with a HER2-0 metastasis (**a**, **c** haematoxylin & eosin (HE) staining; **b**, **d** HER2 IHC). Case 2: a HER2-low (2+) primary tumor with a HER2-positive metastasis (**e**, **g** HE staining; **f**, **h** HER2 IHC). Case 3: a HER2-low (1+) primary tumor with a HER2-low (1+) metastasis (**i**, **k** HE staining; **j**, **l** HER2 IHC). Case 4: a HER2-low (2+) primary tumor with a HER2-low (1+) metastasis (**m**, **o** HE staining; **n**, **p** HER2 IHC)

Figure 3 summarizes the discordance of HER2 status between primary breast cancer and distant metastases, while Fig. 4 shows examples of typical immunohistochemical staining of HER2. The overall HER2 discordant rate was 27.6% (*n* = 27). Among all these changed patients, 81.5% (*n* = 22/27) turned into HER2-0, while 19.5% (*n* = 5/27) became HER2-3+, of which 2 cases had positive FISH results due to the original score of HER2 2+ and others were lack of FISH test (Fig. 3a). And 4 cases with HER2-3+ in metastases were HER2-2+ in primary sites except 1 case being HER2-1+. The percentage of HER2 discordant cases did not differ among primary tumor phenotypes. In detail, as shown in Fig. 3b, cases with HER2 discordance in HR-positive and triple-negative breast cancers were 24.7% and 38.1% (*p* = 0.222). There was no significant difference in HER2 discordance rate when divided primary BC into Luminal A, Luminal B and TNBC (*p* = 0.392) (Fig. 3c). And the rate of HER2 discordance differed among different tumor sizes of the primary BC (*p* = 0.042), with lower rates observed for T1 tumors (≤ 2 cm) (9.1%), followed by T2 tumors (2.0 cm–5.0 cm) (27.3%) and T3 tumors (> 5.0 cm) (66.7%) (Fig. 3d).

### Effect of metastatic tumor sample on HER2 status evolution

**Fig. 5:**
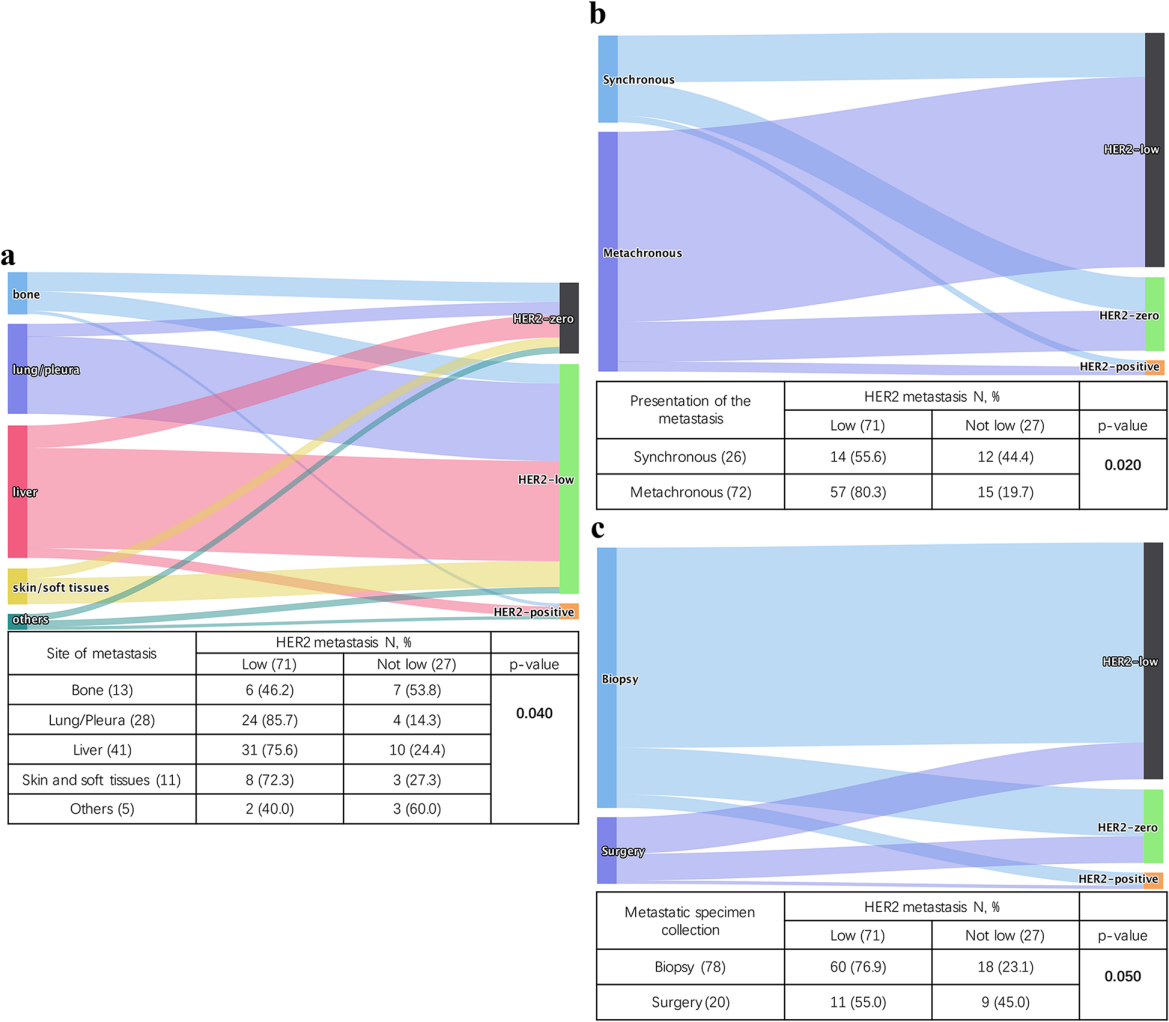
Effect of metastatic tumor sample on HER2 status evolution. The diagram shows the evolution of the HER2 status between primary tumors and metastases stratified by site of metastasis (**a**) (Fisher’s exact test), presentation of metastases (**b**) (Chi-square test) and metastatic specimen type (**c**) (Chi-square test). N, number

In our cohort, distant metastatic sites included lung/pleura (28.6%, *n* = 28), liver (41.8%, *n* = 41), bone (13.3%, *n* = 13), skin and soft tissue (11.2%, *n* = 11), and others (5.1%, *n* = 5). As shown in Fig. 5a, the HER2 discordant rate notably differed among sites of metastases (*p* = 0.040). In detail, higher rates were observed in other sites (60.0%) and bone (53.8%), and lower rates were observed in skin/soft tissue (27.3%), liver (24.4%) and lung/pleura (14.3%). For the 5 cases of other sites of metastases, two metastasized to rectum and posterior peritoneum separately, and remained HER2-low in metastases. Two cases metastasized to ovary and uterine appendages, and became HER2-0 in metastases. The last one metastasized to parietal lobe and became HER2-3+ in metastases. And there are 5 cases in our cohorts which had double metastatic sites, as shown in Table 2. For metastases with discordant HER2 status, the data of the first metastatic site was selected for analysis.

**Table 2:** Cases with double metastatic sites

Case number	Interval time of metastases	Metastatic sites	HER2 status of metastases
1	0 months	Soft tissue	0
		Nasopharynx	
2	24 months	Lung	0
		Bone	1+
3	36 months	Lung	1+
		Liver	
4	20 months	Bone	1+
		Liver	3+
5	24 months	Soft tissue	2+/FISH-
		Liver	

HER2 discordance rate was also influenced by the presentation of the metastasis as synchronous (up to 6 months following the primary diagnosis) or metachronous (*p* = 0.020). Synchronous metastases had higher HER2 discordance rate (Fig. 5b). Moreover, HER2 discordance rate also differed according to type of metastatic BC sample (Fig. 5c). In detail, biopsy included core needle biopsy, endoscopic biopsy and incisional biopsy, and the discordance rate was 23.1% when metastatic BC was assessed on biopsies with being 45.0% on surgical samples, instead (*p* = 0.050).

### Evolution of HER2 IHC score from primary sites to metastases

**Fig. 6:**
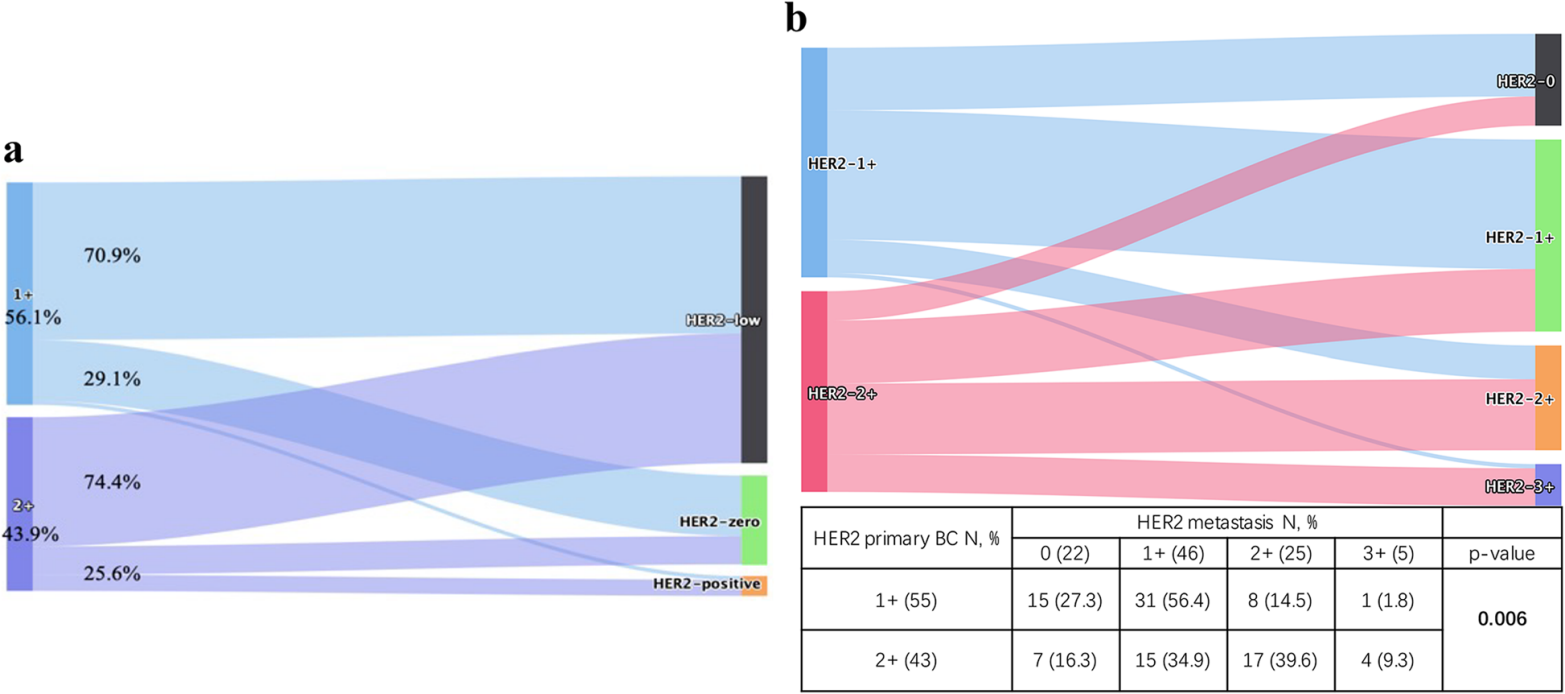
The evolution of the HER2 IHC score from primary breast cancer to metastases. **a** Evolution of HER2 expression according to primary tumor HER2 IHC score. **b** Evolution of the HER2 IHC score from primary breast cancer to metastases (Fisher’s exact test)

Between HER2-low primary BC with different HER2 IHC score, the discordance rate of metastases had no significant difference. In particular, discordant rate of HER2 status was 29.1% (*n* = 16/55) and 25.6% (*n* = 11/43) when primary BC was HER2-1+ and HER2-2+, respectively (*p* = 0.700) (Fig. 6a). When only comparing IHC score of HER2 in metastases, however, the rate of HER2 discordance was significantly lower in HER2-1+ primary BC cohorts (*p* = 0.006) (Fig. 6b). Among HER2-1+ cases, 56.4% (*n* = 31/55) stayed consistent in metastases, while 27.3% (*n* = 15/55) converted to HER2-0 with 14.5% (*n* = 8/55) and 1.8% (*n* = 1/55) switching to HER2-2+ and HER2-3+, respectively. Among HER2-2+ cases, only 39.6% (*n* = 17/43) had concordant HER2 status in metastases, while 16.3% (*n* = 7/43) and 34.9% (*n* = 15/43) converted to HER2-0 and HER2-1+ separately, and the percentage of patients switching to HER2-3+ was 9.3% (Fig. 6b).

### Survival analysis

**Fig. 7:**
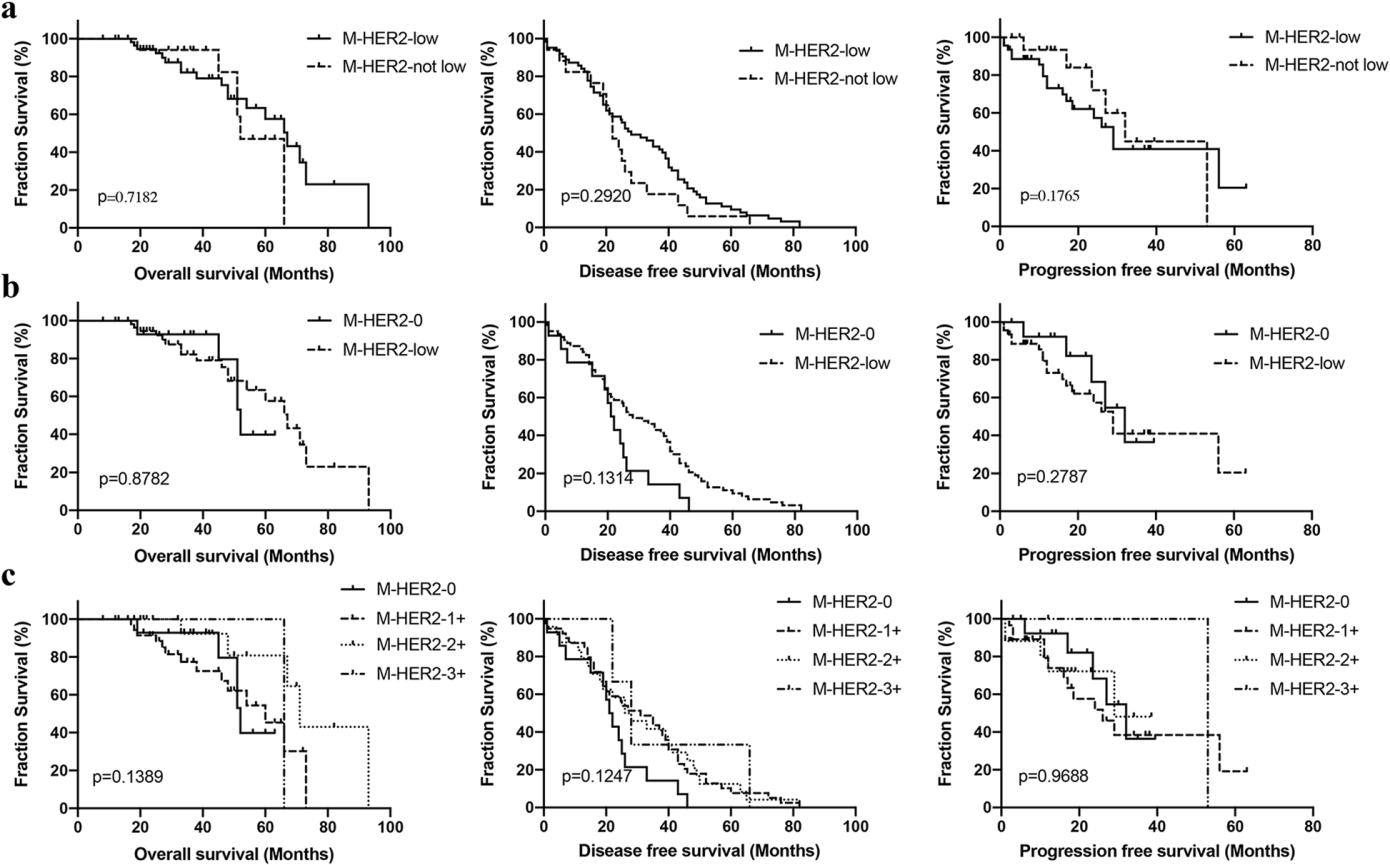
Kaplan–Meier survival curve for overall survival, disease free survival and progression free survival. **a** Disease outcomes according to HER2 status in metastases. **b** Disease outcomes were compared between HER2-0 and HER2-low metastatic samples. **c** Disease outcomes were compared among different HER2 IHC scores in metastases

We compared OS, DFS and PFS separately according to HER2 status in metastatic sites. We didn’t observe significant difference among metastases with different HER2 status (Fig. 7a). Median OS was 67 months for HER2-low metastatic tumors compared with 52 months for HER2-not-low metastatic tumors (*p* = 0.718), median DFS was 28 months for HER2-low metastatic tumors compared with 22 months for HER2-not-low metastatic tumors (*p* = 0.292), and median PFS was 29 months for HER2-low metastatic tumors compared with 32 months for HER2-not-low metastatic tumors (*p* = 0.1765). The results didn’t change much when excluding cases that metastases of which changed to HER2-3+ (Fig. 7b). We also compared OS,DFS and PFS between metastases with different HER2 IHC score, and no significant difference was found, either (Fig. 7c).

## Discussion

Due to the new ADCs, HER2-low breast cancer is getting much more attention and becoming a new entity, which elevated the clinical complexity of BC. HER2 status changes between primary and metastatic breast cancer have been reported in some studies with inconsistent results (see Additional file 1). Different from previous studies about HER2-negative BCs [18, 19], our analysis investigated the changes of HER2 expression in patients with HER2-low BC and their distant metastases.

In our cohort, we included HER2-low BC patients with distant metastases, and the proportion of patients with HER2-1+ primary BC was higher than that of HER2-2+, which was consistent with reported evidence [20, 21]. Also, we found a significantly higher percentage of HR+ BC (78.6%) than that of TNBC, which was similar with previous studies which reported that proportion of HR+ cases was higher than TNBC among HER2-low breast tumors [21–23].

Mainly, we wanted to examine how HER2-low status evolved from primary sites to distant metastases, and the overall HER2 discordant rate was 27.6%. In particular, 22.4% of HER2-low primary tumor switched to HER2-0 in metastases, which was consistent with a previous study that 22% of HER2-low primary tumor switched to HER2-0 in the advanced stage [19]. Interestingly, we found an association between T stages of primary tumors and altered HER2 expression in metastases. In particular, the higher the T stage of the primary BC, the higher the rate of HER2 discordance. And the HER2 discordant rate was higher in surgical specimen than biopsy for the specimen type of metastasis. These may be explained – at least in part – by the intratumor heterogeneity of HER2. Previous studies have already proved that both HER2 expression and HER2 gene amplification have intratumor heterogeneity [24, 25], and in HER2-low BCs, the degree of intratumor heterogeneity is obviously higher [26]. For tumor with larger size, there may be higher frequency of coexistence of multiple tumor-cell subpopulations with distinct HER2 status, and this may influence the discordance between primary tumor and its metastases. And although, currently core-needle-biopsy is believed to be acceptable to get enough samples for HER2 expression assessment [27], our findings suggest that it may be necessary for surgical resection or at least multi-point biopsy of metastases in clinical practice to obtain accurate HER2 information. Similarly, higher intratumor heterogeneity in primary tumor, which has been reported being associated with breast cancer progression and worse prognosis [26, 28], may also lead to shorter metastatic intervals and resulted in higher discordance rate in synchronous metastases.

It’s worth to be noted that our cohort showed the rate of HER2 evolution varied between different metastatic sites, and the highest discordance rate was shown in bone metastases while lung metastases shown the lowest HER2 discordant rate. Although our analysis focused on HER2-low primary tumor and the matched metastases, these results were similar with previous study that included HER2-0 and HER2-low primary tumor and the matched recurrent tumor [18]. However, controversial results were also reported [19]. And the possible role of the decalcification process on the results may be doubted. Firstly, we checked the records of all bone metastases, and all samples were not decalcified. And there also has been reported that there is a significant correlation between ERBB2 mRNA and HER2 protein levels in bone metastases [29]. So, we tend to believe that the data on bone metastases is reliable. And one possible explanation for the higher discordance rate in bone metastases is that the biopsies of bone metastases were more likely to yield insufficient tissue for examination than other metastatic sites [30]. And relatively higher discordance rate in bone metastases were also reported in similar studies. A study about HER2-negative BC [18] and a review including HER2-negative and HER2-positive BC [31] both reported bone metastases had higher discordance rate.

Another worth-noting observation is that although it was reported that there were molecular biological differences between HR+ and HR- in people with low HER2 expression [21], the HER2 discordance rate didn’t differ between HR-positive and triple-negative breast cancers in our cohorts, which was inconsistent with the previous study [18]. And we didn’t find any difference of HER2 discordance between HER2-1+ and HER2-2+ patients, but we observed that HER2-2+ patients were less likely to have consistent metastatic HER2 levels than HER2-1+ patients when comparing HER2 IHC scores. This may indicate that more attention is needed for HER2 status in metastases when primary tumor being HER2-2+. And to investigate possible survival differences, we also performed survival analysis. Either OS, DFS or PFS was observed no difference among metastases with different HER2 status, which suggested that there was no prognostic value for HER2 discordance in metastases of HER2-low BC.

Our work has several strengths. Firstly, we rescored all of the HER2 IHC slides according to the latest standard of HER2 diagnosis by ASCO/CAP Guidelines Update [27]. On the one hand, the cut-offs for HER2-zero were downgraded since October 2013 according to the ASCO/CAP HER2 testing guideline [32], while we have cases before 2013 in our cohort. On the other hand, before the concept of HER2-low was proposed, the emphasis was on the distinction between HER2-negative and positive for clinical practice, and there could be errors in the diagnosis of HER2-0 and HER2-1+. So, we think it is necessary to re-evaluate the HER2 IHC score of all cases. Due to limited conditions, we didn’t choose old archival paraffin blocks or stored unstained paraffin slides for re-staining, which may also have the problem of antigenicity loss [33–35]. And considering the fact that positive and negative controls were set in all cases and HER2 IHC scores decreased in only 8 cases after reevaluation, we believe that the fading problem of DAB staining does not affect the reliability of rescored results. In addition, HER2 expression of all the cases was tested by Ventana 4B5 antibody on Ventana BenchMark autostainer using the same automatic staining protocol, with barcode generated by the autostainer on every slide (see Additional files 2 and 3), which ruled out the possible influence of different antibodies on HER2 discordance [36]. However, some limitations need to be emphasized as well. Firstly, patients included in the analysis received heterogenous systemic treatments, which might influence the HER2 status [37] and impair definitive conclusions on survival outcomes. Moreover, we only included patients with a historical primary score of HER2-low expression. This resulted in limited number of our cases, which may lead to some bias in our comparisons. And more HER2-low BCs may be found if we rescored patients with a historical primary score of HER2-0, considering the poor accuracy of HER2 IHC score in 0 and 1+ [38], which might expand our study cohort.

## Conclusions

For primary BCs with HER2-low status, there is a possibility of HER2 status alteration in the metastases. The rate of altered HER2-low expression was different among different metastatic sites, and the discordant rate of bone metastasis was the highest. The discordant rates of HER2 were also different among different T stages of primary BC and different specimen type of metastasis. No difference of HER2 discordance rate was found between HER2-1+ and HER2-2+ patients. No prognostic significance was observed. These data further support development of best practices for identifying patients with HER2-low expression who could benefit from anti-HER2 ADCs.

## Supplementary Information

**Additional file 1.** Literature on HER2 (all scores) changes between primary tumors and metastases [39–47].

**Additional file 2.** Laboratory accreditation certificate by China National Accreditation Service for Conformity Assessment (CNAS).

**Additional file 3.** Assessment of HER2 IHC by Nordic Immunohistochemical Quality Control (NordiQC).

## Data Availability

All data generated or analysed during this study are included in this published article.

## Abbreviations

BC: Breast cancer
HER2: Human epidermal growth factor receptor 2
IHC: Immunohistochemistry
ISH: In situ hybridization
ER: Estrogen receptor
PR: Progesterone receptor
ADCs: Antibody-drug conjugates
ORR: Objective response rate
HR: Hormone receptor
TNBC: Triple negative breast cancer
PFS: Progression free survival
OS: Overall survival
FISH: Fluorescence in situ hybridization
HE: Haematoxylin & eosin
