# Supplementary material for: HER2-low breast cancer: evolution of HER2 expression from primary tumor to distant metastases
Source: BMC Cancer. 2023 Jul 13;23:656. doi: 10.1186/s12885-023-11134-4 (PMC10347880; doi:10.1186/s12885-023-11134-4)
Supplement: Supplementary file 2 — Additional file 2. Laboratory accreditation certificate by China National Accreditation Service for Conformity Assessment (CNAS). [file 12885_2023_11134_MOESM2_ESM.pdf]

China National Accreditation Service for Conformity Assessment

LABORATORY ACCREDITATION CERTIFICATE

(Registration No. CNAS MT0133)

Department of Pathology,

Cancer Hospital Affiliated to Fudan University

(Legal Entity: Cancer Hospital Affiliated to Fudan University)

No.270, Dong'an Road, Xuhui District, Shanghai, China

is accredited in accordance with ISO 15189: 2012 Medical Laboratories-Requirements for Quality and Competence (CNAS-CL02 Accreditation Criteria for the Quality and Competence of Medical Laboratories) for the competence to undertake testing service as described in the schedule attached to this certificate.

The scope of accreditation is detailed in the attached schedule bearing the same registration number as above. The schedule forms an integral part of this certificate.

Effective Date：2022-08-08

Expiry Date：2028-08-07

Signed on behalf of China National Accreditation Service for Conformity Assessment

China National Accreditation Service for Conformity Assessment (CNAS) is authorized by Certification and Accreditation Administration of the People's Republic of China (CNCA) to operate the national accreditation schemes for conformity assessment. CNAS is a signatory of the International Laboratory Accreditation Cooperation Mutual Recognition Arrangement (ILAC MRA) and the Asia Pacific Accreditation Cooperation Mutual Recognition Arrangement (AP AC MRA).
The validity of the certificate can be checked on CNAS website at http://www.cnas.org.cn/english/findanaccreditedbody/index.shtml.
