# Supplementary material for: HER2-low breast cancer: evolution of HER2 expression from primary tumor to distant metastases
Source: BMC Cancer. 2023 Jul 13;23:656. doi: 10.1186/s12885-023-11134-4 (PMC10347880; doi:10.1186/s12885-023-11134-4)
Supplement: Supplementary file 3 — Additional file 3. Assessment of HER2 IHC by Nordic Immunohistochemical Quality Control (NordiQC). [file 12885_2023_11134_MOESM3_ESM.pdf]

NordiQC

Nordic Immunohistochemical Quality Control

Institute of Pathology, Aalborg University Hospital, Ladegaardsgade 3, P.O.Box 561, DK-9100 Aalborg, Denmark

Assessment of HER2 IHC, B31 - individual results

Fudan University Shanghai Cancer Center (1412)

**Epitope**	HER2 IHC
**Assessment**	Optimal

NordiQC has assessed the submitted slides. In general, the assessment is based on staining intensity and distribution in cells expected to be demonstrated, background staining, cross-reactivity, quality of counter-staining and preservation of tissue morphology. Specific criteria for each epitope are described on http://www.nordiqc.org/epitope.php.
Each slide was marked as optimal, good, borderline or poor based on the following criteria:

Optimal: The staining reaction is considered perfect or close to perfect in all of the included tissues.
Good: The staining reaction is considered acceptable in all of the included tissues. However, the protocol settings may be optimized to ensure improved sensitivity or higher signal-to-noise ratio.
Borderline: The staining reaction is considered insufficient because of a generally too weak staining reaction, false negative or false positive staining reaction of one of the included tissues. The protocol should be optimized.
Poor: The staining reaction is considered insufficient because of, e.g., false negative or false positive staining reactions of several of the included tissues. An optimization of the protocol is urgently needed.

Moderate or strong cross reaction (due to the character of the primary antibody) or other false positive staining reaction (e.g. due to endogenous biotin) is not compatible with an optimal result and will usually cause downgrading.
For stains assessed as borderline or poor, comments and recommendations are given to the protocols. Good stains may also be accompanied by comments if specific problems are identified.
Recommended protocols from each staining platform are available at the NordiQC homepage (http://www.nordiqc.org/recommended.php) for comparison. Implementation of NordiQC recommended protocols as well as changes suggested in this letter must be tested carefully in your own laboratory before implementation into diagnostic work. NordiQC do not take any responsibility for consequences of changes in protocols or methods in your laboratory.
In case of a borderline or poor staining result, laboratories may request reassessment of the original stain or a new stain at following run.
